# *CERKL* Knockdown Causes Retinal Degeneration in Zebrafish

**DOI:** 10.1371/journal.pone.0064048

**Published:** 2013-05-09

**Authors:** Marina Riera, Demian Burguera, Jordi Garcia-Fernàndez, Roser Gonzàlez-Duarte

**Affiliations:** 1 Departament de Genètica, Facultat de Biologia, Universitat de Barcelona, Barcelona, Spain; 2 Institut de Biomedicina (IBUB), Universitat de Barcelona, Barcelona, Spain; 3 CIBERER, Instituto de Salud Carlos III, Barcelona, Spain; Laboratoire Arago, France

## Abstract

The human *CERKL* gene is responsible for common and severe forms of retinal dystrophies. Despite intense *in vitro* studies at the molecular and cellular level and *in vivo* analyses of the retina of murine knockout models, CERKL function remains unknown. In this study, we aimed to approach the developmental and functional features of *cerkl* in *Danio rerio* within an Evo-Devo framework. We show that gene expression increases from early developmental stages until the formation of the retina in the optic cup. Unlike the high mRNA-*CERKL* isoform multiplicity shown in mammals, the moderate transcriptional complexity in fish facilitates phenotypic studies derived from gene silencing. Moreover, of relevance to pathogenicity, teleost CERKL shares the two main human protein isoforms. Morpholino injection has been used to generate a *cerkl* knockdown zebrafish model. The morphant phenotype results in abnormal eye development with lamination defects, failure to develop photoreceptor outer segments, increased apoptosis of retinal cells and small eyes. Our data support that zebrafish Cerkl does not interfere with proliferation and neural differentiation during early developmental stages but is relevant for survival and protection of the retinal tissue. Overall, we propose that this zebrafish model is a powerful tool to unveil CERKL contribution to human retinal degeneration.

## Introduction

Retinal dystrophies (RD), the major cause of incurable familial blindness in the Western world, are monogenic disorders characterized by progressive dysfunction of photoreceptor and retinal pigment epithelium (RPE) cells [1]. RD is a group of extremely heterogeneous diseases that show substantial clinical and genetic overlap. Moreover, mutations in a single gene appear to be associated to distinct clinical entities [2], as is the case for *CERKL*, that was initially characterized as an autosomal recessive Retinitis Pigmentosa (RP) gene [3], [4], [5], [6], [7], [8], and later shown to promote Cone-Rod Dystrophy (CRD), a RD disorder associated to a more severe phenotype [9], [10].

Highthroughput technologies have greatly improved our knowledge of the genetic basis of RD. Indeed, more than 180 RD genes have already been reported and this number is constantly increasing (Retnet, https://sph.uth.tmc.edu/retnet/). However, although RD genes are known to be involved in a variety of cellular and molecular processes in the retina, we are still far from understanding the contribution of most of them to the disease. CERKL ranks in this class, as all previous attempts have failed to provide valuable clues to explain its involvement in photoreceptor degeneration.

Human *CERKL* was initially identified as a 13 exon-gene, which encoded a polypeptide of 532 amino acids. This protein shared an integral diacylglycerol kinase (DAGK) signature [3] with Ceramide Kinase (CERK), an ubiquitously expressed paralog with ceramide kinase activity involved in cell survival and proliferation [11]. In CERKL, all the *in vivo* and *in vitro* assays with reported CERK substrates and a variety of lipid mixtures have failed to show any kinase activity [12], [13], [14], [15], [16]. Concerning cell survival, overexpression of CERKL in cultured cells showed protection against apoptosis induced by oxidative stress [14]. Moreover, studies with transfected cell lines have shown a dynamic subcellular localization of CERKL, shifting from the cytoplasm, where the protein is mainly associated to the endoplasmic reticulum and Golgi membranes, to the nucleus [14]. CERKL intracellular traffic regulation seems to be directed by two nuclear localization signals (NLSs) and two nuclear export signals (NESs) [6], [12], [13]. Concerning CERKL localization in the retina, immunohistochemistry on mouse cryosections revealed strong localization in cones, faint in rods, and moderate at the ganglion cell (GCL) and inner nuclear layers (INL) [17], [18], [19].

CERKL performance in the retina has been also approached through an accurate assessment of its transcriptional products in several tissues. Interestingly, in the retina, human and mouse *CERKL* revealed an unexpected high repertoire of mRNA isoforms (>20 isoforms in human and >30 in mouse were validated), which emerged from alternative splicing and additional promoters, among them that of *NEUROD1* gene [17], [18]. The high heterogeneity presumed at the protein level, together with its dynamic subcellular localization probably accounts for the multi-functional character of CERKL.

Animal models, whether natural or transgenic, provide invaluable tools for studies of disease pathogenesis and the identification of therapeutic targets [20]. To date, two mouse models of CERKL have been constructed. The first was obtained by deletion of the alternatively spliced exon 5, where the most prevalent mutation (R257X) is found [21]. The second was generated in our group by the deletion of the proximal promoter and exon 1. Both mouse models were viable and fertile, and did not show gross morphological alterations in the retina. Our targeted *Cerkl* deletion resulted in a knockdown rather than a knockout model, as gene transcription was attained from two previously unreported alternative promoters [19]. Moderate dysfunction was observed in the ganglion and/or amacrine cells, supported by aberrant electroretinographic recordings and increased retinal apoptosis and gliosis, whereas photoreceptor cells showed WT features [19]. The failure to reproduce the human phenotype in the mouse, not unusual in other hereditary retinal disorders, prompted us to explore zebrafish as an alternative model. In this context, *Danio rerio* seems to be an excellent tool to understand the mechanisms of human visual disorders, because human and zebrafish share the main cell types and general structure of the eye. Moreover, zebrafish biology allows ready access to all developmental stages, and the optical transparency of embryos and larvae allow real-time imaging of developing pathologies [22]. The ontogeny of the zebrafish eye begins as an evagination from the developing forebrain around 12 hours postfertilization (hpf), and ocular development is largely completed by 72 hpf, at which time the first visual responses can be detected [23], [24], [25], [26]. In this study we have identified the zebrafish *cerkl* ortholog, studied its expression during development and in the adult tissues, and drawn comparisons with vertebrate species. Besides, we have generated *cerkl* zebrafish knockdowns by morpholino injection and characterized a range of developmental abnormalities in the morphant phenotype, including retinal degeneration and apoptosis-like cell death. Finally, our analyses highlight our model as a simple and amenable tool to analyse CERKL contribution to RD pathogenesis.

## Materials and Methods

### Ethics statement

All procedures were performed according to the ARVO Statement for the Use of Animals in Ophthalmic and Vision Research, as well as the regulations of the Animal Care facilities at the University of Barcelona. The study was approved by the Ethics Committee for Animal Experimentation (CEEA) of the University of Barcelona. When needed, animals were sacrificed with excess of anaesthetic MS222, following the approved protocols.

### Animal handling, tissue dissection and preparation of the samples

Zebrafish (*Danio rerio*) were maintained at 28.5°C on a 14-hour light/10-hour dark cycle. The transgenic strain *ath5:GFP* was a kind gift from Carolina Minguillón. Fertilized eggs were obtained and grown in incubators, and embryos were staged as described [27], [28]. Specific tissues and organs were dissected from adult zebrafish and immediately frozen in liquid nitrogen.

### Identification of CERKL orthologs

The human CERKL isoform 1 (NM_201548.4) amino acid sequence was used as a query for a BLASTp search (http://www.ncbi.nlm.nih.gov). Protein sequences from several species were compared running a CLUSTALW2 alignment (www.ebi.ac.uk). Conservation of CERKL across different species was evaluated with the Jalview program (version 2.7).

### RNA-seq expression analysis

Available RNA-seq data on zebrafish developmental stages and adult tissues was used to quantify *cerkl* expression according to the previously defined cRPKM value [29]. The reported RNA-seq data used in this study are shown in Table S1.

### RNA extraction and RT-PCR

RNAs from a pool of zebrafish, frog and chicken embryos at different stages of development or from different tissues of adult specimens were extracted using the RNeasy Mini or Micro Kit (Qiagen, Valencia, CA), following the manufacter's instructions. RT-PCR assays were carried out with the Transcriptor High Fidelity cDNA Synthesis Kit (Roche Diagnostics, Indianapolis, IN), using 200 ng of total RNA. For semi-quantitative analysis, the cDNA was amplified according to standard protocols using GoTaq polymerase (Promega, Madison, WI). The level of expression and characterization of different isoforms was performed using a forward primer located in the 5′UTR and a reverse primer in the 3′UTR (Table S2). β-*actin*, *ODC* and *Gapdh* were used for normalization in zebrafish, frog and gallus samples, respectively (see primer sequences in Table S2). All PCR products were resolved on agarose gel electrophoresis and sequenced.

### Cloning and overexpression of zebrafish CERKL in cultured cells

The full-length zebrafish *cerkl* transcript was amplified from adult retina oligo-dT cDNA using specific primers carrying *Bam*HI and *Xho*I restriction enzyme sites (see primer sequences, Table S2). The cDNA was inserted into a modified version of the pcDNA3.1 vector (Clontech Laboratories, Inc., Mountain View, CA) that adds a C-terminal hemagglutinin (HA) tag.

For protein expression, COS-7 cells were seeded and transfected using Lipofectamine 2000 reagent (Invitrogen Life Technologies, Carlsbad, CA), according to the manufacturer's protocol. After 48 h, immunolocalization was performed as previously described [14] incubating the cells with 1∶275 anti-HA mouse monoclonal antibody (Covance, Princeton, NJ) followed by 1∶300 AlexaFluor 488-conjugated anti-mouse secondary antibody (Invitrogen Life Technologies). Slides were counter-stained with 1∶5000 DAPI (Roche Diagnostics, Indianapolis, IN) nuclear blue dye in PBS for 15 min. All preparations were mounted in Flouprep medium (BioMérieux, Craponne, France) and analyzed by confocal microscopy (SP2, Leica Microsystems, Wetzlar, Germany).

### Histology and in situ hybridization

Embryos and adult zebrafish eyecups were fixed in 4% paraformaldehyde (PFA). For cryosections, embryos and adult eyecups were rinsed in sucrose at 4°C (successive incubations at 20% for 30 min, 30% for 30 min and 40% sucrose for 12 h) and then were embedded in O.C.T (Tissue-Tek, Sakura Finetech, Torrance, CA) and sectioned at −17°C. *In situ* hybridization on whole-mounts and cryosections were performed as previously described [30], [31] using digoxigenin (DIG)-labelled RNA sense and antisense probes (see primer sequences in Table S2). The BM Purple AP Substrate (Roche Diagnostics, Indianapolis, IN) reagent was used. Sections were cover-slipped with Fluoprep (Biomérieux, Craponne, France) and photographed using a Leica DFC Camera connected to a Leica DM IL optic microscope (Leica Microsystems).

### Morpholino and mRNA injection

To knockdown ZF*cerkl*, we used two morpholino antisense oligonucleotides (MOs) targeting the acceptor splice site at the boundary of intron 3 and exon 4 (acMO, 5′-TCTCAGTGACTGTGGAAAAGAAAGA-3′) and the donor splice site at the boundary of exon 9 and intron 9 (doMO, 5′-TAACCATACTCACAAATGTCTCCTC-3′). A standard control MO (coMO, 5′-CCTCTTACCTCAGTTACAATTTATA-3′) was also used. All the MOs were designed and synthesized by GeneTools (Philomath, OR). Eight nanograms of each MO were air pressured injected into 1 to 4-cell embryos. For the phenotypic rescue, human cDNA was cloned into the pCS2 vector. *In vitro* transcription of synthetic capped mRNA was performed using a capped RNA transcription kit (SP6 mMESSAGE mMACHINE; Ambion, Austin, TX) following the manufacturer's instruction. Two nanoliters of MO or mixed MO/mRNA was injected into each 1 to 4 cell-stage embryo. The final concentrations of MO and mRNA were 200 µM and 400 ng/ µl, respectively.

### Haematoxylin and eosin staining and immunohistochemistry

Retina cryosections of 72 hpf embryos were haematoxylin and eosin stained under standard conditions. For immunohistochemistry, 14 µm sections were recovered on poly-lysine covered slides, dried for 1 h, washed in PBS (3× 10 min), and blocked in blocking solution (PBS containing 3% sheep serum, 1% BSA and 0.3% Triton X-100) for 60 min at room temperature (RT). Incubation with peanut agglutinin (PNA) conjugated to Alexa Fluor 647 (40 mg/ml; Invitrogen Life Technologies) and the primary antibody mouse anti-rhodopsin (1∶500, Abcam, Cambridge, MA) was performed overnight at RT in blocking solution. Sections were rinsed three times in PBS again, followed by incubation with Alexa-Fluor568 goat anti-rabbit as secondary antibody (1∶300, Invitrogen Life Technologies). Nuclei were stained with DAPI (Roche Diagnostics), sections were mounted in Fluoprep medium (Biomérieux) and analyzed by confocal microscope (SP5, Leica Microsystems). For eye measurements, 14 µm thick cryosections were examined under the microscope and imaged. Eye size was taken from the anterior to the posterior edge using the Fiji software. Significant differences between groups were analyzed by the Student's *t*-test.

Apoptotic cells in the retina cryosections of 72 hpf morphants were detected by immunofluorescence using anti-active Caspase-3 as primary antibody (1∶200, BD Pharmingen, San Jose, CA) and Alexa-Fluor568 goat anti-rabbit as secondary antibody (1∶300, Invitrogen Life Technologies) following the protocols already described. For apoptosis quantification, nuclei were counted using the Fiji software.

## Results

### Homology search and cloning of the zebrafish *cerkl*

To identify zebrafish *cerkl (*ZF*cerkl*), a BLAST search of the Ensembl zebrafish database was performed using the human CERKL protein isoform 1 sequence (NP_963842). A single copy of the zebrafish *cerkl* gene was detected, encompassing 195 kb on chromosome 9. The genomic region of ZF*cerkl* shows conserved synteny over more than 1,8 Mb with the human *CERKL* locus on chromosome 2, and both largely share the same gene order and transcriptional orientation, with the only exception of a chromosomal inversion encompassing 4 genes (*dnajc*, *frzb*, *nckap1*, *dusp19*) located 5′ upstream of *cerkl* (Fig. S1).

**Figure 1:**
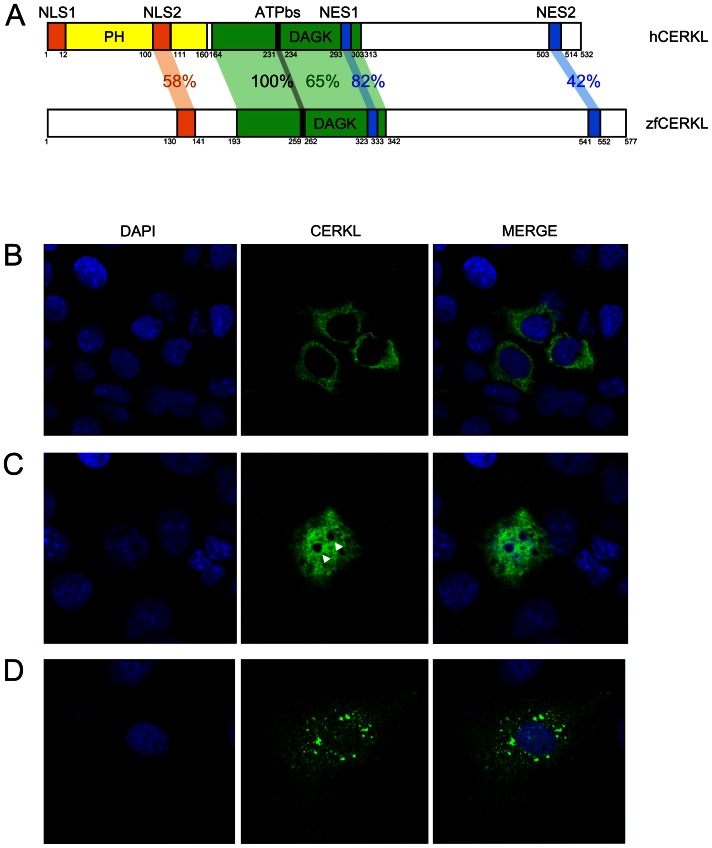
Human and zebrafish CERKL protein domains. (**A**) The reported hCERKL (NP_963842) protein domains described by either sequence homology (PH, pleckstrin homology; DAGK, diacylglycerol kinase and ATPbs, ATP binding site domains) or by functional analysis (NLS, nuclear localization signals; NES, nuclear export signals) and their conservation in zebrafish is shown in percentage of identity. (**B**–**D**) ZFCerkl-HA shares with human CERKL the dynamic subcellular localization in COS-7 transfected cells, shifting from the cytoplasm to the nucleus. Nuclei were stained with DAPI. Images correspond to individual optical sections. Photographs were at×63 magnification. (**B**) In most cells, ZFCerkl shows a uniform distribution in the cytosol and is absent from the nucleus. (**C**) Some cells per field showed localization of Cerkl in both, the cytosol and the nucleus, with clear exclusion from the nucleoli (white arrow). (**D**) Rarely, Cerkl contributes to cytosolic aggregates.

The predicted zebrafish transcript encompasses 13 exons, spans approximately 2 kb and encodes a protein of 577 amino acids, the latter showing 59% identity and 82% similarity with the human counterpart. CERKL alignments between zebrafish and human revealed 65% identity between the DAGK domains (Fig. 1A). The ATP binding site (ATPbs) GGDG motif contained in this domain, already described in CERK, was fully conserved not only in zebrafish but also in 5 vertebrate species (Fig. S2). Concerning the nuclear localization and export signals (NLS and NES, respectively), the human NLS1 sequence (MPWRRRRNRVSA) was not conserved in zebrafish, neither in the rest of the species analysed, whereas NLS2 (SVKLKRRCSVKQ) showed 58% identity, with preservation of all but one (L) of the five key residues (underlined). The NES1 (LHIIMGHVQL) and NES2 (LMEVASEVHIRL) domains of human and zebrafish CERKL displayed 82% and 42% identities, respectively. The *in silico* predicted pleckstrin homology (PH) domain, essential for CERK localization, translocation, and enzymatic activity in human, encoded in exons 1 and 2 [32], showed very low conservation among vertebrate species. Remarkably, the multi-species comparison of CERKL protein sequences revealed two previously unidentified highly conserved regions, one in exon 7 (hCERKL, 318–352 aa), and the other encompassing exons 10 and 11 (hCERKL, 400–448 aa), with unassigned function so far (Fig. S2).

On the basis of these predictions, specific primers were designed (Table S2) to clone zebrafish *cerkl* cDNA by RT-PCR into a modified version of the pcDNA 3.1 vector that adds a C-terminal hemagglutinin (HA) tag. The construct was transfected into COS-7 cells, and an anti-HA immunodetection was performed. The ZFCerkl protein shared with hCERKL the dynamic subcellular localization, shifting from the cytoplasm (where is found with a uniform pattern or in aggregates) to the nucleus. Remarkably, in contrast with the human homolog, ZFCerkl showed clear exclusion from the nucleoli (Fig. 1B–D).

### Regulated expression of vertebrate *cerkl* orthologs during embryogenesis and in adult tissues

**Figure 2:**
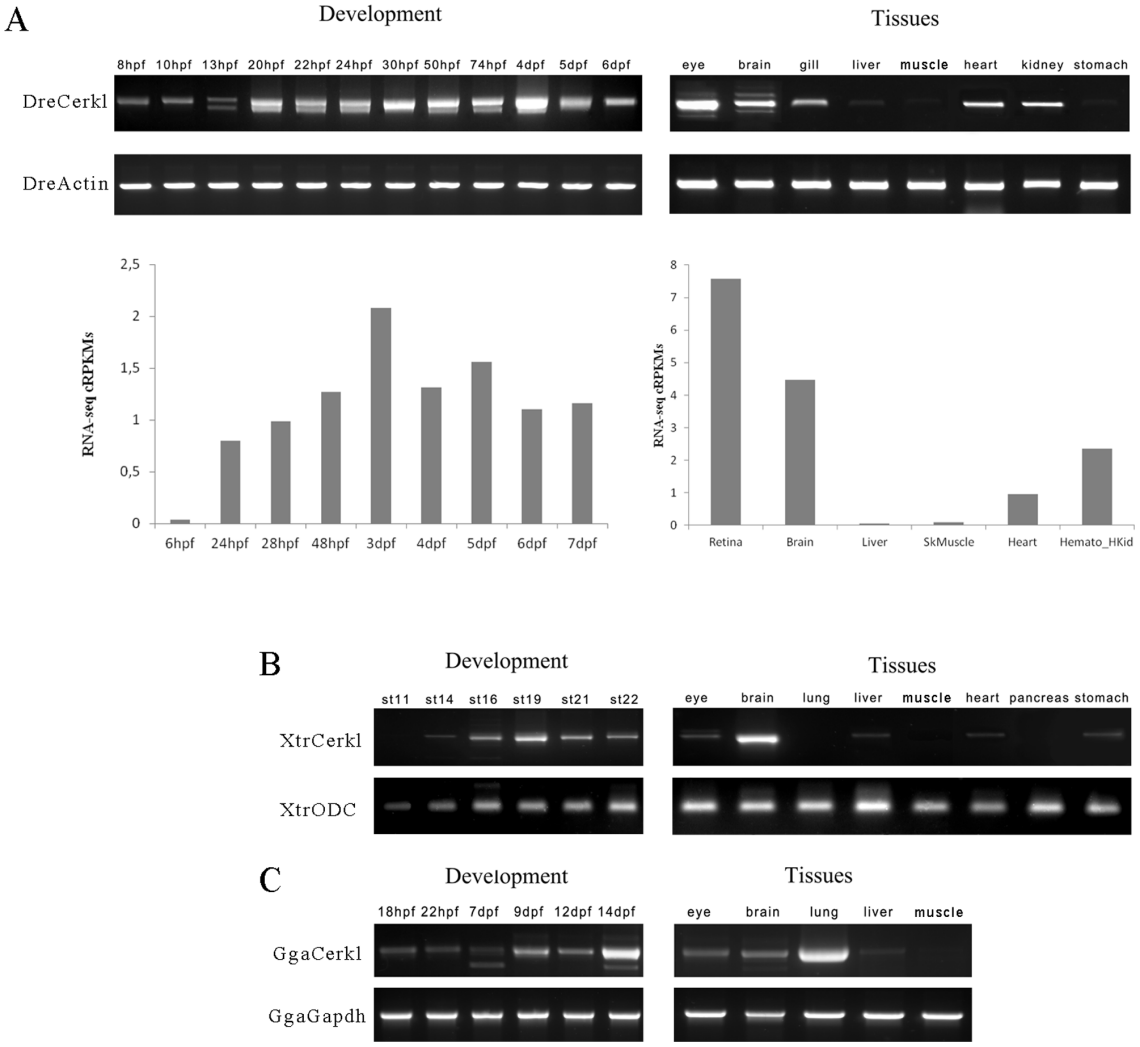
Expression of *cerkl* transcripts during embryonic development and adult tissues. (**A**) Temporal and spatial expression of zebrafish (Dre) *cerkl* assessed by RT-PCR (top) and RNA-seq data retrieved from databases (bottom) at different developmental stages and adult tissues. (**B and C**) Expression of *cerkl* in developmental stages and tissues of frog (Xtr) and chicken (Gga). hpf, hours post-fertilisation; dpf, days post-fertilization; st, stage.

The temporal and spatial expression pattern of ZF*cerkl* during embryogenesis was examined by semi-quantitative RT-PCR (Fig. 2A, see Table S2 for primer sequences). Expression was faint at early developmental stages, from 75%-epiboly (8 hpf) up to the 8-somite stage (13 hpf) embryos, followed by two marked increases in gene expression around the 22-somite (20 hpf) and 30 hpf stages, just when the optic cup starts forming the neural retina [33]. Moreover, the expression persisted during development up to 6 days post fecundation (dpf), reaching a maximum at 4 dpf. Adult tissue expression was examined in total RNA from eye, brain, gill, fin, heart, liver, muscle and stomach. Expression was highest in eye, moderate in brain, heart and kidney, and low in gills. Our RT-PCR semiquantitative analysis was supported by available zebrafish RNA-seq data (Fig. 2A) [34], [35], [36], [37], [38].

We then aimed to assess whether *cerkl* tissue-specific regulation and developmental expression was evolutionary conserved among other vertebrate species: *Gallus gallus* and *Xenopus tropicalis*. In agreement with mammals, *cerkl* expression in both species was mainly detected in eye and brain (Fig. 2B and C) [17], [18], which suggested a conserved role in the eye and the anterior central nervous system (CNS). Interestingly, high levels of transcription were also detected in the lung of *Gallus.* Further work should clarify if this is a case of *cerkl* co-option in the avian lineage.

**Figure 3:**
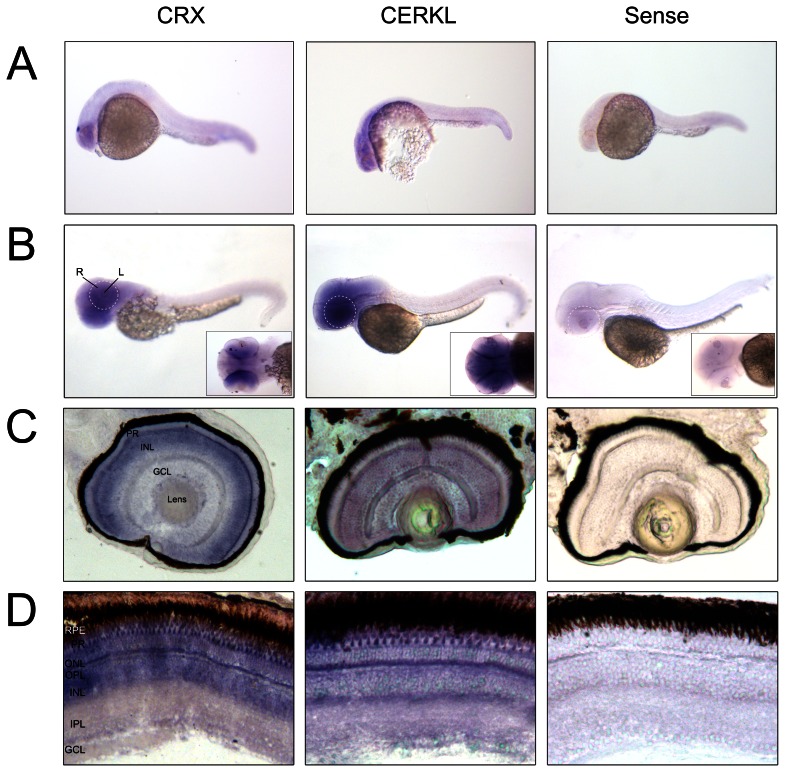
*Cerkl in situ* hybridization on embryo and adult zebrafish. (**A and B**) Whole-mount RNA *in situ* hybridization analysis showing *cerkl* expression in the retina and brain of embryos at 24 and 50 hpf. R, retina; L, lens. (**C and D**) *In situ* hybridization on zebrafish retina cryosections of 72 hpf embryos and adult tissue. *Cerkl* expression is detected in the three nuclear layers of the embryo retina, whereas adult expression appears in the inner segment of the photoreceptors and some cells located at the basal layer of the INL. Positive control (antisense *CRX*), strongly labels the inner photoreceptor segment and inner nuclear layer. The negative control was performed with sense *cerkl*. RPE, retinal pigment epithelium; PR, photoreceptor; ONL, outer nuclear layer; OPL, outer plexiform layer; INL, inner nuclear layer; IPL, inner plexiform layer; GCL, ganglion cell layer.

Tissue specific expression in zebrafish was also assessed in whole embryos and in embryonic and adult retinas by *in situ* hybridization (ISH) (Fig. 3). *Crx* antisense and *cerkl* sense riboprobes were used as positive and negative controls, respectively. Faint *Cerkl* expression was already detected at 24 hpf by whole-mount ISH (Fig. 3A). Fifty hpf embryos showed *cerkl* expression restricted to the anterior region and, particularly, in the eye and brain (Fig. 3B). To further characterize the retinal expression pattern, ISH on cryosections was performed. Our data revealed strong hybridization signal at 72 hpf embryos in the three nuclear layers (Fig. 3C), whereas adult expression concentrated in the inner segment of photoreceptors and staining was not homogeneous along the inner nuclear and ganglion cell layers (Fig. 3D).

### Alternative splicing and the NeuroD1 promoter among vertebrates

**Figure 4:**
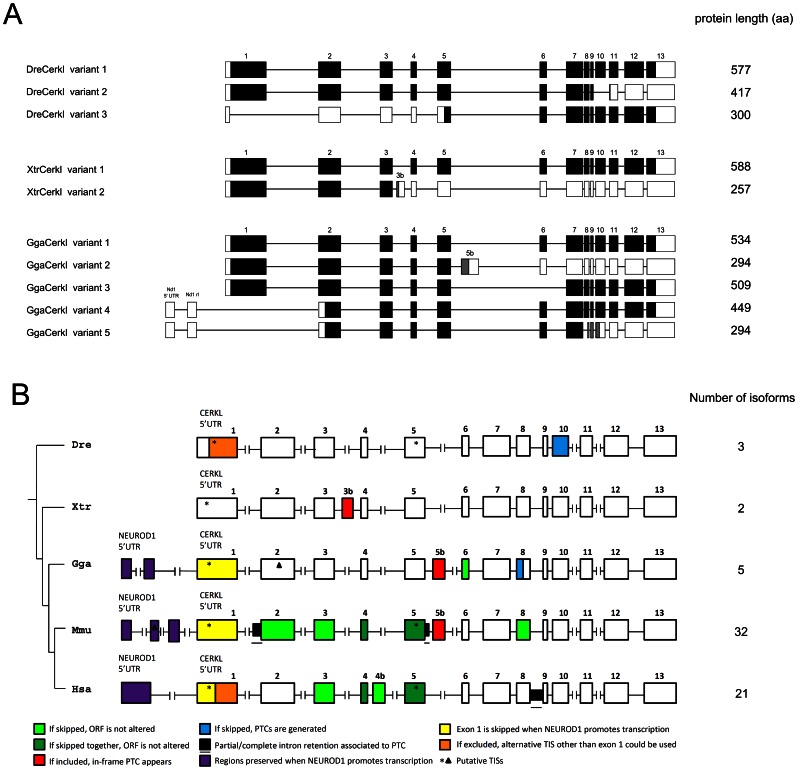
Alternatively spliced *cerkl* isoforms in vertebrates. (**A**) Scheme of the *cerkl* mRNA transcripts identified in zebrafish (Dre), frog (Xtr) and chicken (Gga). Exons are boxed and the coding sequence (CDS) for each isoform, considering the largest ORF, is shown in black. Grey boxes represent alternative ORFs. Protein length (in aa) is indicated (right column). (**B**) Schematic view of *CERKL* gene structure as well as the translational impact of all alternative exons (AEs) detected in *Danio rerio* (Dre), *Xenopus tropicalis* (Xtr), *Gallus gallus* (Gga), *Mus musculus* (Mmu) and *Homo sapiens* (Hsa). Exons preserved in all protein isoforms are shown by empty boxes. AEs are colored: light green, if when skipped, the ORF is not altered; dark green for those that maintain the ORF when skipped together; red, if they contain an in-frame stop codon and thus, when preserved, produce a truncated C-terminus protein. In blue, exonic sequences that, when skipped, the ORF generates a premature stop codon; black underlined flattened boxes indicate partial/complete intron retention associated to truncated peptides, purple depicts the regions preserved when *NEUROD1* promotes transcription. The latter isoforms lead to whole exon 1 depletion and subsequent loss of the conventional initiator methionine. In the canonical human and zebrafish *CERKL* first exon two donor splice sites are contained, one at the 3′end of the exon, and the other in the middle (the boundary shown in orange). When this second splice site is used, protein translation begins in exon 5. Asterisks show initiator methionines validated in human only but conserved among vertebrates. A methionine in exon 2 of *Gallus gallus* that could be used to initiate translation when exon 1 is skipped is depicted by a triangle. The number of *CERKL* isoforms in each species is indicated (right column). PTC: premature termination codon; TIS: translation initiation site.

To assess and compare the diversity of *cerkl* mRNA isoforms in vertebrate, 45-cycle PCR reactions were performed using primers located in exons 1 and 13 of *cerkl* of *Danio rerio, Gallus gallus* and *Xenopus tropicalis* (Fig. 4A). Keeping in mind that some AS events and additional alternative promoters could have escaped our experimental approach, 3 *cerkl* mRNA variants were identified in zebrafish, 2 in *Xenopus tropicalis* and 3 in *Gallus gallus*. In zebrafish, variant 1 was the major isoform and encompassed all the exons; variant 2 skipped exon 10 and encoded a C-terminus truncated protein without the NES2 domain; and variant 3, adult eye-specific, was generated from a novel splice donor site within exon 1. In the later form, protein synthesis would start at a conserved methionine in exon 5, as already described in human [18], and truncation would affect the full NLS2 and ATP binding site signatures, and a fraction of the DAGK domain. In *Xenopus tropicalis*: variant 1 was the main isoform with all the canonical exons, and variant 2, eye-specific, incorporate an alternative exon (3b), between exons 3 and 4, containing an in-frame stop codon. In *Gallus gallus*: variant 1, the main isoform, comprised all the annotated exons; variant 2 included a stop-containing exon (5b, between exons 5 and 6) and encoded a C-terminal truncated protein; and variant 3, skipped exon 6 and generated an in-frame shortened peptide.

We also aimed to characterize *CERKL* expression in the three analysed species from the upstream transcriptional start site (TSS) of *NEUROD1* [18], [19], a transcriptional factor involved in photoreceptor development [39]. RT-PCR eye cDNA assays with specific primers spanning the *neuroD1* 5′UTR and exon 2 or 13 of *cerkl* showed that NeuroD1 promoted *cerkl* expression in chicken, but not in zebrafish or frog. The use of the alternative promoter generated a novel 5′UTR exon, between *neuroD1* and *cerkl Gallus* genes, and generated two new isoforms (named 4 and 5) (Fig. 4A). If translated, the peptides would show truncations at the N-terminus, as the canonical methionine in exon 1 was skipped and translation could be initiated from a species-specific methionine in exon 2. A general picture of the alternative exons and their impact on the predicted ORF in human, mouse, chicken, frog and zebrafish *CERKL* is shown in Fig. 4B.

### Validation of *cerkl* morpholinos

**Figure 5:**
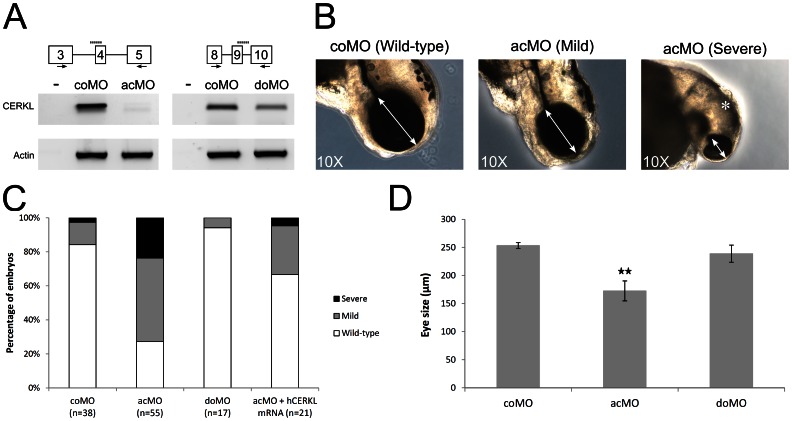
Effects of ZF*cerkl* silencing in eye development. (**A**) Two morpholinos targeting an acceptor (acMO) and donor splice site (doMO) of ZF*cerkl* were used. The morpholino targeting site is depicted by a discontinuous line. To assess the knockdown effect, a RT-PCR analysis of control and ZF*cerkl* morphants injected with 8 ng of MO was performed (primers used are depicted with arrows). Silencing with acMO was almost complete, when compared with *cerkl* expression in 72 hpf control MO-injected embryos (coMO), whereas in doMO animals the WT spliced isoform was decreased by 35%. β*-Actin* was used for normalization. (**B**) acMO-injected embryos displayed either small eye phenotype (named mild phenotype) or very small eye, small head, and body and curved tail (named severe phenotype). White double-head arrows denote diameter of the eyes. * denotes small and curved head in severe-phenotype morphants. (**C**) Phenotype frequency of morphants. The *cerkl*-knockdown phenotype was rescued when human *CERKL* mRNA was co-injected with acMO. n, number of individuals. (**D**) Eye size (in diameter) was measured in at least ten independent embryos from each group. Data were analyzed by *t*-test and are presented as mean ± SEM. ** P<0.001. Mean eye size was 253.4 µm for control, 172.7 µm for acMO and 239 µm for doMO morphants.

To evaluate whether the knockdown of ZF*cerkl* caused a retinal defect, two antisense morpholinos (MOs) that targeted all mRNA isoforms were used, acMo and doMO (see the Material and Methods section), and a standard negative control (coMO). In order to assess splicing blockage by ZF*cerkl* MOs, we performed RT-PCR analyses from a pool of 10–12 embryos at different stages (24, 30, 52 and 72 hpf) using specific primers flanking the target region (Fig. 5A, black arrows). In the case of acMO, almost complete depletion (85%) of *cerkl* transcripts was attained, whereas doMO blockage was less intense, only reaching the 35%. The poor blocking ability of doMO was taken to mimic the heterozygous condition of human carriers.

To further investigate the putative *ZFcerkl* aberrant splice products produced by acMO and doMO blocking, semi-quantitative RT-PCR assays were devised. Nor exon skipping neither intron retention could be identified using specific primers located at the flanking exons or introns of each MO targets. Assuming that transcripts from the targeted alleles would have skipped exon 4 or exon 9, or retained intron 3 or intron 9, in acMO and doMo, respectively, a premature translation-termination codon (PTC) would appear in all cases. To verify if the PTC-containing mRNAs-ZFCerkl were degraded via the nonsense-mediated mRNA decay (NMD) surveillance mechanism, another RT-PCR assay was carried out with two pairs of primers located in exons far away from the original morpholino targets (see Table S2 for primer sequences). Expression levels were in accordance with those reported for the first pair of primers in each case, thus supporting NMD transcript depletion (Fig. S3). Indeed, this is in agreement with late reports showing that NMD effectors are deeply involved in zebrafish embryonic development and survival [40].

### ZF*cerkl* suppression in retinopathy

In accordance with our previous observations showing greater blocking ability for the acMO, microinjection of 8 ng MO into 1 to 4 cell-stage embryos resulted in morphogenesis defects in 73% of the animals, whereas no mutant phenotypes were observed neither with doMO-, nor with coMO- injected embryos, from stage 24 hpf to 5 dpf. First evidences of distortions in the visual system were detected at 48 hpf. The main traits were eye size reduction with overall structure preservation and clear boundaries between the lens and the neural retina in acMO embryos. By 72 hpf, around 70% of the acMO morphants showed the small eye phenotype (“mild” form), and some (23%) also exhibited small heads, markedly curved body axes and short tails (“severe” form) (Fig. 5B). Phenotypic rescue was attained after co-injection of the acMO and the mRNA encoding human wild type *CERKL* (Fig. 5C). Analyses of the diameter length of the eye on histological sections indicated a 29% reduction of the acMO treated embryos compared with coMO animals (Fig. 5D).

**Figure 6:**
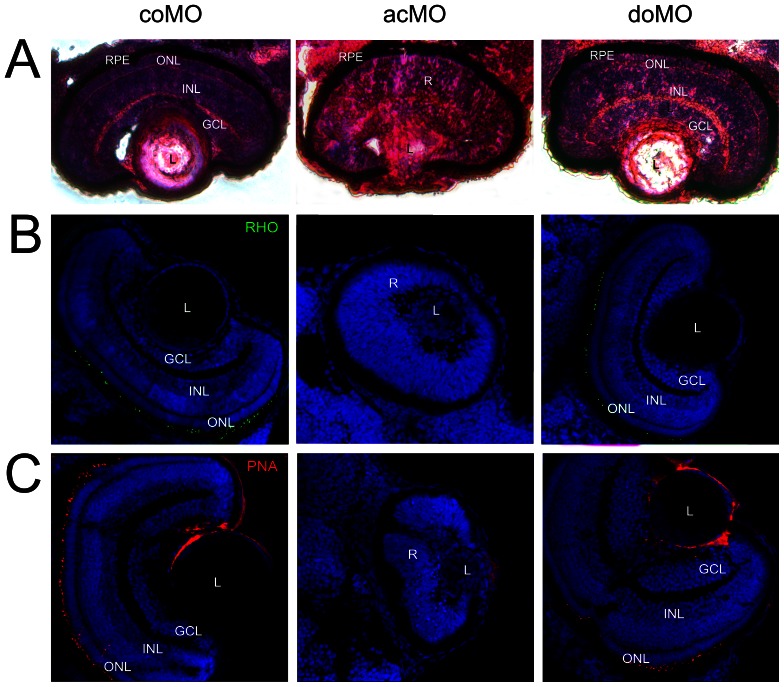
Eye histology in control and ZF*cerkl* morpholino-injected embryos at 72 hpf. (**A**) Zebrafish eye sections were stained with H&E. Control morphants (coMO) showed normal retinal lamination with three cell layers (GCL, INL, and ONL). In “mild” acMO-injected embryos, lamination did not occur and the three layers were not visible. The retinal pigment epithelium (RPE) developed normally in control and ZF*cerkl* morphants. (**B**) Immunostaining with anti-rhodopsin and (**C**) anti-PNA identified rod and cone outer segments, respectively, in control and doMO morphants. Outer segments were absent in acMO morphants. Nuclei were stained with DAPI. Some nonspecific staining was seen in the lens when stained with PNA. Photographs were at×40 magnification.

To investigate the effect of *cerkl* knockdown on retinal development, histological and immunological analysis of 72 hpf retinas of ZF*cerkl*-deficient morphants and controls were carried out. By this stage, the outer segment of photoreceptors is normally formed. In our case, the acMO-injected morphants showed defective lamination as the three retina cell layers (GCL, INL and outer nuclear layer, ONL) were absent, whereas wild type lamination was observed in doMO and control embryos (Fig. 6A). Moreover, the outer segments of rod and cone photoreceptors (detected with anti-rhodopsin and anti-PNA, respectively) were absent in acMO embryos (Fig. 6B, C). The development of the retinal pigmented epithelium (RPE) was unaffected. Another relevant trait of the acMO morphants was the abnormal lens morphology, possibly related to the retardation of ocular development because of *cerkl* knockdown.

### Knockdown of ZF*cerkl* leads to cell death

**Figure 7:**
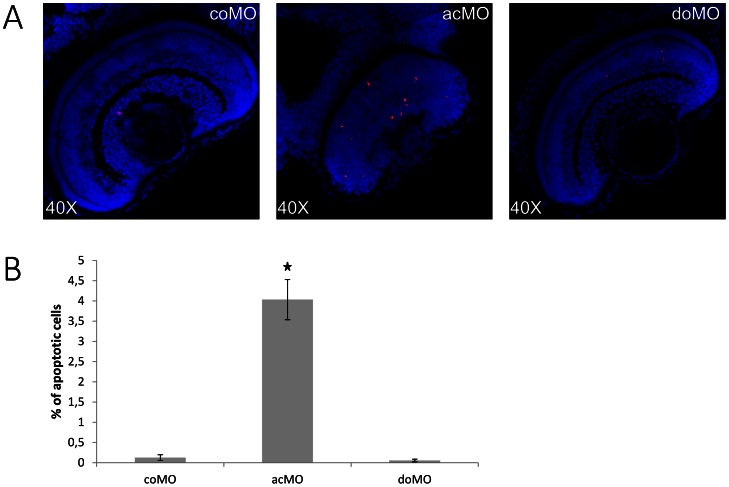
Increased cell death in ZF*cerkl* morpholino (acMO)-injected embryos. (**A**) Immunodetection of apoptosis by anti-active Caspase-3 in retina cryosections of 72 hpf control (coMO) and ZF*cerkl* morpholino-injected embryos (acMO and doMO). Caspase-3-positive cells (shown in red) increased in acMO morphants. Nuclei were stained with DAPI. (**B**) The percentage of apoptotic cells in each retina within a single cell layer was quantified in four independent embryos from each group, plotted and analysed by *t*-test. Data are presented as mean ± SEM. * P  =  0.005.

The aberrant eye phenotype could be due to increased cell death or reduced proliferation. Immunodetection of Caspase-3 (a marker of the first stages of apoptosis [41]) was used to assess cell death in single optical sections (Fig. 7A). Positive cells were scored in 4 independent embryos from each group. Our results revealed a 16-fold increase of cell death in acMO embryos (Fig. 7B) and supported increased apoptosis of retinal cells.

### Cerkl does not contribute to retinal cell proliferation and early differentiation

**Figure 8:**
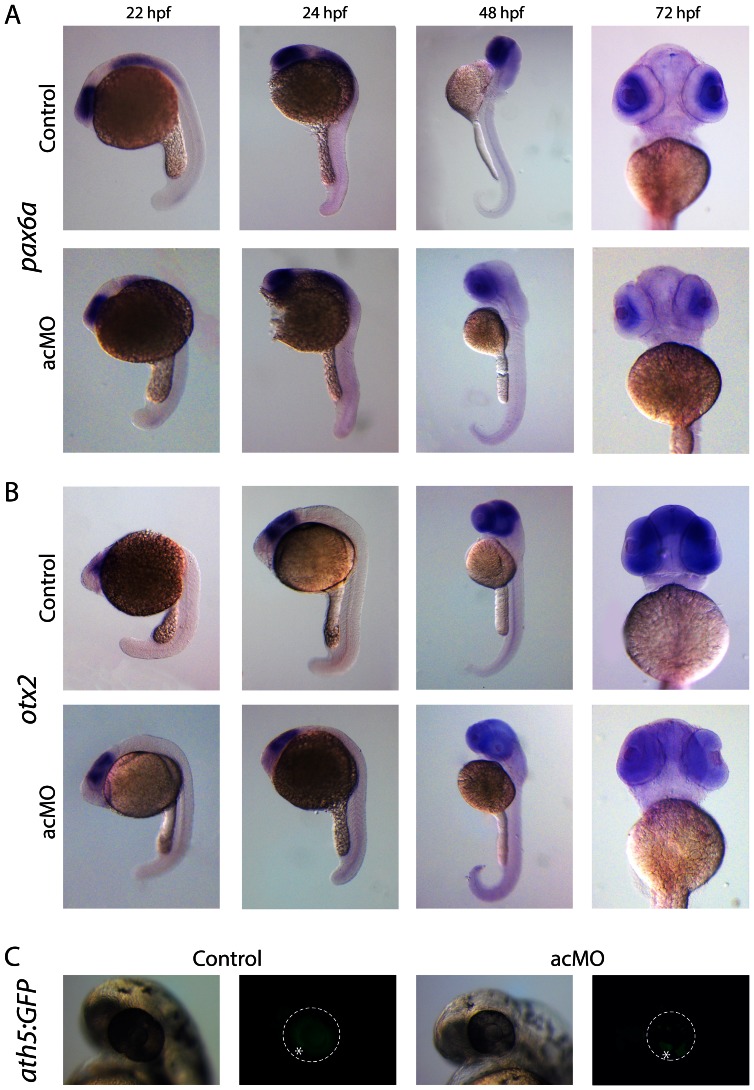
Expression of retina cell markers in acMO-injected embryos at early developmental stages. (**A and B**) At 22 and 24 hpf, the spatiotemporal pattern of *pax6a* (A) and *otx2* (B) in acMO-injected animals was similar to that of controls: *pax6a* was detected in the forebrain, hindbrain, spinal cord and eye, and *otx2* in the eye and midbrain. By 48 and becoming more evident at 72 hpf, acMO embryos exhibited a marked reduction in the expression of both markers. (**C**) The expression of the *ath5* transcription factor was assessed *in vivo* in acMO-injected embryos of the transgenic *ath5:GFP* strain. At 48 hpf, the wave of ath5 expression, which prefigures the wave of retinal ganglion cell genesis, filled the central and peripheral retina of control embryos, whereas the pattern appeared delayed and disorganized in the acMO morphants, although RGC genesis was not fully abolished. * denotes the ventronasal patch of RGC genesis.

To assess if the acMO phenotype in zebrafish was due to defective cell proliferation or differentiation during early retinal development or a secondary degeneration process, whole-mount ISH assays with early retinal markers *pax6a* and *otx2* were performed (Fig. 8A and B). *Pax6a* contributes to the control of cell proliferation, maintenance of the retinogenic potential of the retina progenitor cells, and amacrine cell fate specification [42], whereas *otx2* is a key regulatory gene for retinal photoreceptor determination [43]. Our results indicated that at early stages, 22 and 24 hpf, when in the optic vesicle all cells are still proliferating, the expression pattern of both, *pax6a* and *otx2,* was unaffected in the acMO morphants. As expected, *pax6a* was detected in the developing forebrain, hindbrain, spinal cord, and eye, and *otx2* in the eye and midbrain. At 48 and even more evident at 72 hpf, when all cells are postmitotic, the acMO embryos exhibited a considerable decrease in hybridization intensity compared to the controls, treated and stained under the same conditions. The distribution of differentiation markers in the acMO morphants was further analyzed using the *ath5:GFP* transgenic strain. *Ath5* is a transcription factor expressed in a wave-like pattern that prefigures the wave of the retinal ganglion cell (RGC) genesis, the first cell type to differentiate in the vertebrate retina, which has been previously reported as affected in our mouse model *Cerkl* ^−/−^. The wave of *ath5* expression and RGC differentiation in zebrafish occurred during the second day post-fertilization, starting in the ventronasal patch and spreading from there to the rest of the nasal and central retina [28]. By 48 hpf, the RGC wave filled the central and peripheral retina in control embryos, whereas it was delayed in acMO morphants (Fig. 8C). Hence, our results suggested that *cerkl* was not essential for driving the RGC differentiation wave, as the ventronasal patch was formed and the wave started spreading. However, in agreement with the above results in cell death, the delay and disorganization in RGC neurogenesis supported Cerkl contribution to cell survival, and suggested that the small eye phenotype and the defective retina lamination were a consequence of a secondary degeneration process.

## Discussion

After intense studies, the role in photoreceptor degeneration of *CERKL*, a gene causing autosomal recessive RP and CRD [3], has remained elusive up to now. We have gathered data showing that CERKL protects cells form oxidative stress [14] and, lately, in our *Cerkl* ^−/−^ murine model, in favor of a consistent and notable decrease of the retinal sphingolipid content particularly, the glucosyl/galactosylceramide species [16]. Although these results could be suggestive of CERKL and glucosylceramide involvement against oxidative stress, we still are far from understanding CERKL role in pathogenesis. In this work, we have aimed to gain new insights into CERKL retinal function generating a zebrafish knockdown model.

Transfection of ZFCerkl in cultured COS-7 cells supported the dynamic nuclear-cytoplasmic localization (Fig. 1) and the NLS2 major role in directing nuclear trafficking as previously described in human [13]. The role of CERKL in the nucleus is still unclear and does not seem to be related to the transcriptional regulation of sphingolipid-related genes [16]. However, the physiological relevance of NLS2 is stressed by its structural conservation across species and R106S, a mutation-causing RP, precisely located in this domain [6].

We have shown that high transcriptional complexity of CERKL in mammals (namely human and mouse) arise from the combination of tissue-specific promoters (among them *NEUROD1*) and alternative splicing [17], [18], [19]. Although the regulatory meaning of AS is largely unknown, even sometimes considered as background spliceosomal noise [44], [45], [46], it could also be related to the fine-tuning of key biological processes. Interestingly, recent work has revealed that adaptive novelties have arisen through changes in AS regulation, as ganglion-specific splicing of *TRPV1* underlies infrared sensation in vampire bats [47]. Thus, we aimed to assess *cerkl* transcript diversity in zebrafish, as well as in frog and gallus, and compare them with those reported for mouse and human. Our data showed that the full length (13 exons) coding region was the only isoform shared, and that AS events were seldom conserved in *CERKL*. In terms of exon cassettes, exons 3, 4 and 5 were alternatively spliced only in human and mouse, which supported an evolutionary novelty acquired at some point in the mammalian lineage. Concerning the identification in mouse and chicken of alternative exons between exon 5 and 6 (5b), both bearing a premature stop codon, the convergent inclusion of introns seems the most probably scenario, although an homologous origin could not be fully discarded. Our data are in agreement with the low level of conservation of AS events between vertebrate groups, especially those involving reading frame disruption [48], [49]. Interestingly, the use of NEUROD1 as an alternative promoter for *CERKL* expression appears to be restricted to the amniote species analysed (Fig. 4). Concerning translation, the shorter N-terminal Cerkl protein isoform of zebrafish is the truncated species that mostly resembles the corresponding size variant of mammals.

*Cerkl* morpholino knockdown treatment in wild-type zebrafish embryos clearly affected eye size (Fig. 5) and retinal morphology. At 72 hpf, morphant retinas were defective in lamination and the photoreceptors lacked the outer segments, normally present at this stage (Fig. 6). The number of apoptotic retinal cells was significantly increased in Cerkl-deficient embryos. Cell death did not appear to affect other ocular structures, as cornea and RPE, which highlighted that *cerkl* knockdown effects were restricted to the retina. Similar defects have also been observed for genes encoding transcription factors (e.g., math5, NR2E3) [50], [51], cell cycle regulators (e.g., Cdkn1b/c, cdk5) [52], and photoreceptor ciliary proteins (e.g., RPGR, RP2, TOPORS, BBS9) [53], [54], [55], [56]. Whether the shared phenotype reveals functional overlaps or secondary effects of retinal neurodegeneration, remains to be elucidated. The analysis of different early retinal development markers in acMO morphants suggested that Cerkl might contribute to cell survival but not to cell proliferation or differentiation during early retina development (Fig. 8), in agreement with the reported anti-apoptotic effects reported in cell cultures and animal models [14], [19], [57].

*CERKL* mutations causing RP and CRD disorders are inherited as an autosomal recessive trait. Unlike the phenotype observed in zebrafish *cerkl*-knockdown embryos, patients do not exhibit developmental abnormalities, and neurodegeneration begins at the second-third decade of life. In addition to a dose-dependent effect, another plausible explanation for the lack of developmental defects in humans may be an increased level of complexity, redundancy or robustness in the retinal gene network of mammals that allows for compensation during embryonic development, but fails to maintain a proper function in adulthood.

In conclusion, we propose that although human, mouse and zebrafish *CERKL* phenotype features could be partially dose-dependent, further experiments are needed to identify the functional relevance of each isoform and their individual contribution to the pathogenic threshold. Moreover, the species-specific differences observed deserve further analysis at the temporal and cell-specific level. Interestingly, the availability of a zebrafish model is a powerful tool to elucidate how *cerkl* depletion results in the occurrence of apoptotic cell death after defective retinal lamination and photoreceptor outer segment formation, and provides new scenarios to understand human retinal degeneration.

## Supporting Information

Figure S1 **Syntenic organization of the** ***CERKL*** **genomic region.** Schematic view of the structure and gene organization of the 1.8 Mb genomic locus encompassing *CERKL* in *Homo sapiens* (Hsa), *Xenopus tropicalis* (Xtr) and *Danio rerio* (Dre). The discontinuous lines in the *Xenopus* locus represent the end of the scaffold. Conserved genes are shown in color, while empty arrows depict the end of the syntenic region. Two chromosomal rearrangements are shown in the compared region: a tandem duplication of the TTN gene in zebrafish, located at the right border, and a chromosomal inversion encompassing 4 genes (at the left boundary, framed in red). Concerning the inverted segment, human and *Xenopus tropicalis* share gene order and orientation, suggesting that the chromosome rearrangement took place after the split of tetrapod and teleost lineages. Ancestral condition is unknown, as basal vertebrate genome assemblies are not available.
(TIF)

Figure S2 **Conservation of CERKL across different species.** Accession numbers for the amino acid sequence of each species are: NP_963842, human (*Homo sapiens*); XP_002799006, macaque (*Macaca mulatta*); XP_002712274, rabbit (*Oryctolagus cuniculus*); NP_001041641, mouse (*Mus musculus*); XP_002932061, frog (*Xenopus tropicalis*), XP_421973, chicken (*Gallus gallus*); NP_001082943, zebrafish (*Danio rerio*).
(TIF)

Figure S3 **Validation of cerkl morpholinos.** Transcriptional products obtained with the following sets of primers: (**A**) exons 3-5 and 11-13 for acMO samples, and (**B**) exons 8-10 and 11-13 for doMO samples. The comparable decrease in band intensity suggests transcript depletion in both cerkl morphants.
(TIF)

Table S1 **Zebrafish RNA-seq data.** The reported RNA-seq data showing tissue resource, accession number and name of the study are indicated.
(DOCX)

Table S2 Primer sequences used for gene expression, cloning and *in situ* hybridization. The sequences of all the primers used are shown.
(DOCX)
