# Supplementary figures and images for: CERKL Knockdown Causes Retinal Degeneration in Zebrafish
Source: PLoS One. 2013 May 9;8(5):e64048. doi: 10.1371/journal.pone.0064048 (PMC3650063; doi:10.1371/journal.pone.0064048)

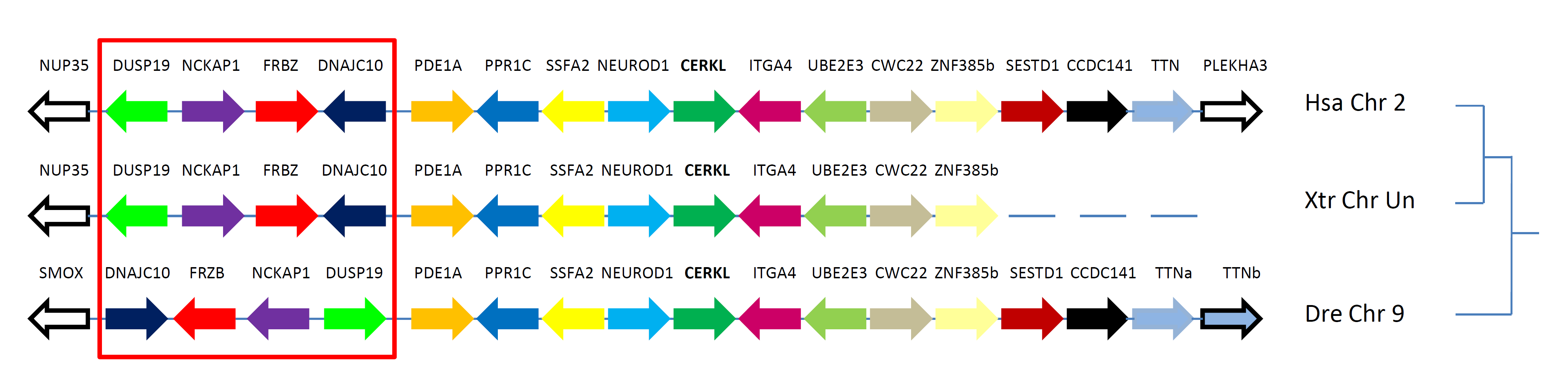

Supplement: Figure S1 — Syntenic organization of the CERKL genomic region. Schematic view of the structure and gene organization of the 1.8 Mb genomic locus encompassing CERKL in Homo sapiens (Hsa), Xenopus tropicalis (Xtr) and Danio rerio (Dre). The discontinuous lines in the Xenopus locus represent the end of the scaffold. Conserved genes are shown in color, while empty arrows depict the end of the syntenic region. Two chromosomal rearrangements are shown in the compared region: a tandem duplication of the TTN gene in zebrafish, located at the right border, and a chromosomal inversion encompassing 4 genes (at the left boundary, framed in red). Concerning the inverted segment, human and Xenopus tropicalis share gene order and orientation, suggesting that the chromosome rearrangement took place after the split of tetrapod and teleost lineages. Ancestral condition is unknown, as basal vertebrate genome assemblies are not available. (TIF) [file pone.0064048.s001.tif]

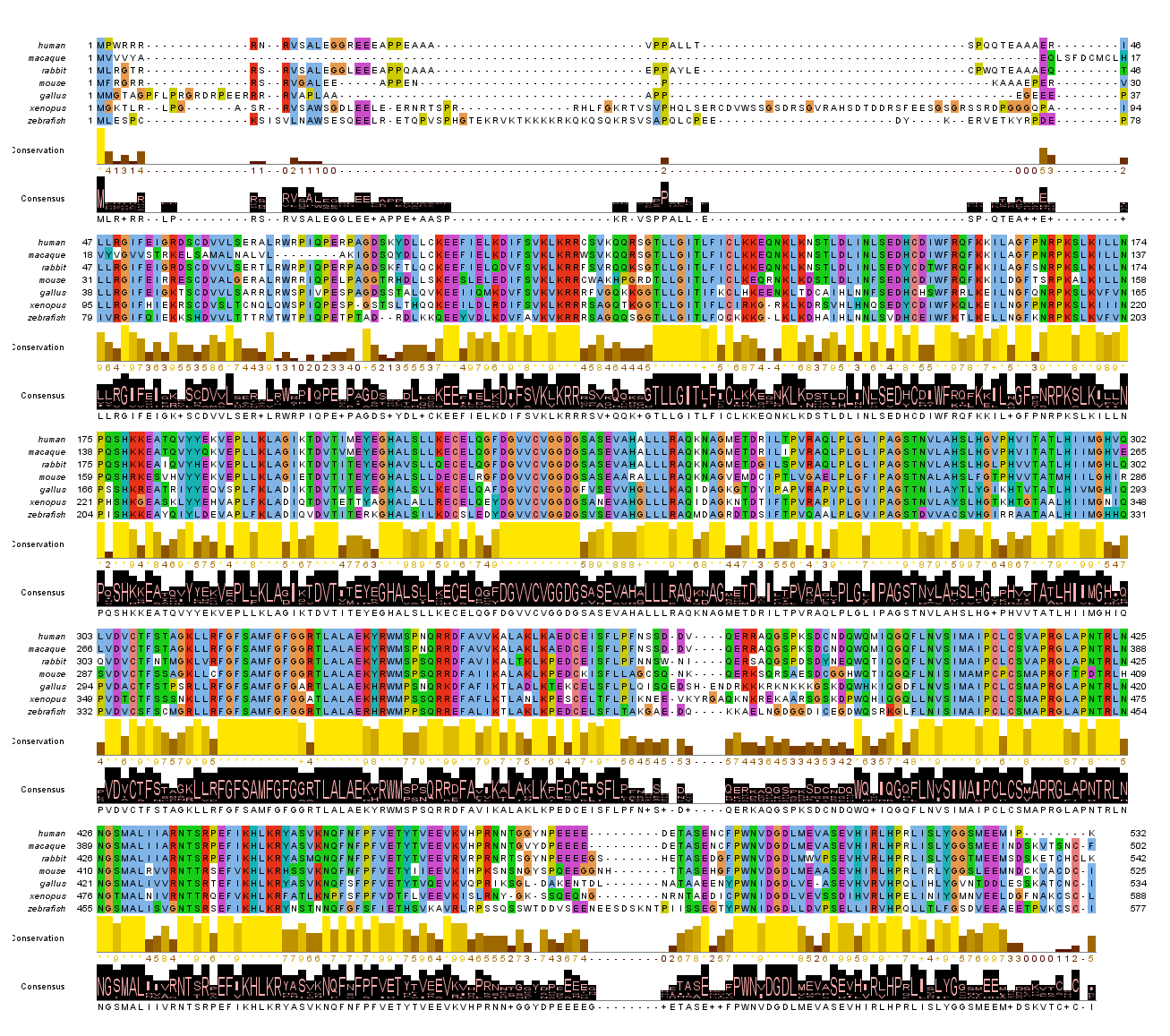

Supplement: Figure S2 — Conservation of CERKL across different species. Accession numbers for the amino acid sequence of each species are: NP_963842, human (Homo sapiens); XP_002799006, macaque (Macaca mulatta); XP_002712274, rabbit (Oryctolagus cuniculus); NP_001041641, mouse (Mus musculus); XP_002932061, frog (Xenopus tropicalis), XP_421973, chicken (Gallus gallus); NP_001082943, zebrafish (Danio rerio). (TIF) [file pone.0064048.s002.tif]

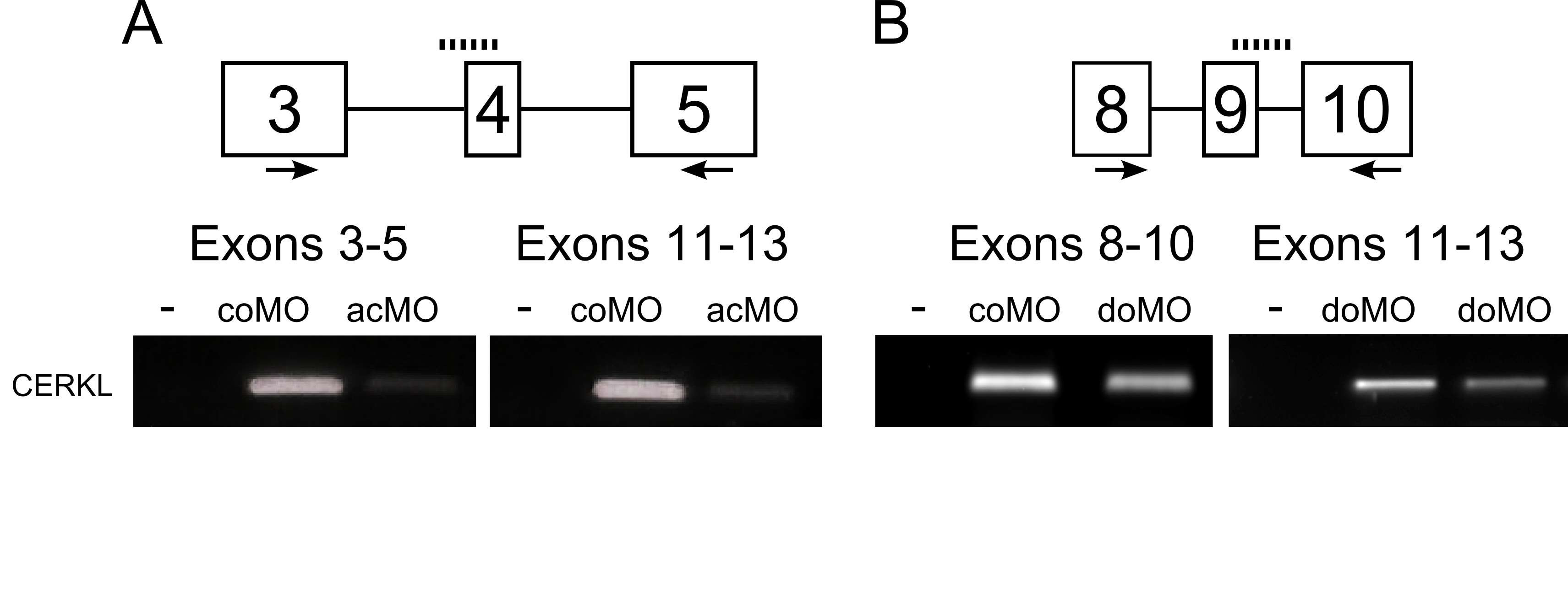

Supplement: Figure S3 — Validation of cerkl morpholinos. Transcriptional products obtained with the following sets of primers: (A) exons 3-5 and 11-13 for acMO samples, and (B) exons 8-10 and 11-13 for doMO samples. The comparable decrease in band intensity suggests transcript depletion in both cerkl morphants. (TIF) [file pone.0064048.s003.tif]
