# Supplementary material for: CERKL Knockdown Causes Retinal Degeneration in Zebrafish
Source: PLoS One. 2013 May 9;8(5):e64048. doi: 10.1371/journal.pone.0064048 (PMC3650063; doi:10.1371/journal.pone.0064048)
Supplement: Table S1 — Zebrafish RNA-seq data. The reported RNA-seq data showing tissue resource, accession number and name of the study are indicated. (DOCX) [file pone.0064048.s004.docx]

| **Table S1.** Zebrafish RNA-seq data | | |  |
| --- | --- | --- | --- |
| Tissues | Accession | Study | Reference paper |
| Brain | ERX009448 | Sanger_zebrafish_sequencing (ERP000447) |  |
|  | ERX009449 | Sanger_zebrafish_sequencing (ERP000447) |  |
|  | ERX013540 | Sanger_zebrafish_sequencing (ERP000447) |  |
| Heart | ERR023145 | Sanger_zebrafish_sequencing (ERP000447) |  |
|  | ERR023150 | Sanger_zebrafish_sequencing (ERP000447) |  |
|  | ERR035546 | Sanger_zebrafish_sequencing (ERP000447) |  |
| Kidney | ERR023146 | Sanger_zebrafish_sequencing (ERP000447) |  |
|  | ERR023149 | Sanger_zebrafish_sequencing (ERP000447) |  |
|  | ERR035547 | Sanger_zebrafish_sequencing (ERP000447) |  |
| Skeletal muscle | ERR145636 | ERP001552 | 36 |
|  | ERR145638 | ERP001552 | 36 |
|  | ERR145651 | ERP001552 | 36 |
|  | ERR145631 | ERP001552 | 36 |
|  | ERR145632 | ERP001552 | 36 |
|  | ERR145647 | ERP001552 | 36 |
| Retina | SRR514028 | GSE38786 | 34 |
|  | SRR514029 | GSE38786 | 34 |
| Liver | SRR392106 | SRP009841 | 38 |
|  | SRR392108 | SRP009841 | 38 |
|  | SRR392109 | SRP009841 | 38 |
|  | SRR392110 | SRP009841 | 38 |
|  | SRR392111 | SRP009841 | 38 |
|  | SRR402758 | SRP009841 | 38 |
| 6hpf | ERR022485 | ERP000400 |  |
| 24hpf | ERR003993 | ERP000016 |  |
|  | ERR003994 | ERP000016 |  |
|  | ERR022486 | ERP000400 |  |
| 28hpf | SRR372798 | GSE32898 | 35 |
|  | SRR372799 | GSE32898 | 35 |
| 48hpf | ERR003998 | ERP000016 |  |
|  | ERR022487 | ERP000400 |  |
|  | SRR372800 | GSE32898 | 35 |
|  | SRR372801 | GSE32898 | 35 |
|  | SRR519720 | GSE38575 | 37 |
|  | SRR519721 | GSE38575 | 37 |
| 3dpf | ERR004002 | ERP000016 |  |
|  | ERR022488 | ERP000400 |  |
| 4dpf | SRR519723 | GSE38575 | 37 |
|  | SRR519724 | GSE38575 | 37 |
| 5dpf | ERR022482 | ERP000400 |  |
|  | ERR022483 | ERP000400 |  |
|  | SRR372802 | GSE32898 | 37 |
| 6dpf | SRR519728 | GSE38575 | 37 |
|  | SRR519729 | GSE38575 | 37 |
|  | SRR519730 | GSE38575 | 37 |
| 7dpf | SRR519733 | GSE38575 | 37 |
|  | SRR519734 | GSE38575 | 37 |
|  | SRR519736 | GSE38575 | 37 |
