# Supplementary material for: CERKL Knockdown Causes Retinal Degeneration in Zebrafish
Source: PLoS One. 2013 May 9;8(5):e64048. doi: 10.1371/journal.pone.0064048 (PMC3650063; doi:10.1371/journal.pone.0064048)
Supplement: Table S2 — Primer sequences used for gene expression, cloning and in situ hybridization. The sequences of all the primers used are shown. (DOCX) [file pone.0064048.s005.docx]

**Table S2.** Primer sequences used for gene expression, cloning and *in situ* hybridization

| **Name** | **Forward primer sequence (5' → 3')** | | **Name** | **Reverse primer sequence (5' → 3')** | |
| --- | --- | --- | --- | --- | --- |
| **Gene expression** | | | | | |
| Dre Actin Fw | | TCAGCCATGGATGATGAAAT | Dre Actin Rv | | GGTCAGGATCTTCATGAGGT |
| Dre 5’ UTR Fw | | GGAGACGCGAGGAACTTCCT | Dre 3’ UTR Rv | | TTCTGTGTATGTTTATTAAGTTAAG |
| Xtr ODC Fw | | CAGCTAGCTGTGGTGTGG | Xtr ODC Rv | | CAACATGGAAACTCACACC |
| Xtr 5’ UTR Fw | | ACTTAGCGAGCGCTGCGATG | Xtr 3’ UTR Rv | | CGTCCAGCTCTTCAACGTTC |
| Gga Gapdh Fw | | GGGCTCATCTGAAGGGTGGTGCTA | Gga Gapdh Rv | | GTGGGGGAGACAGAAGGGAACAGA |
| Gga 5’ UTR Fw | | AGCTGCGACGTGGTGCTGAG | Gga 3’ UTR Rv | | CGAACATGAACTTCTGATGC |
| **Cloning** | | | | | |
| *Bam*HI Fw | | CGGGATCCGGAGACGCGAGGAACTTCCT | *Xho*I Rv | | CCGCTCGAGTCAGATACAGCTGCATTTCACC |
| **NeuroD1 - Cerkl** | | |  | |  |
| Dre Fw | | CATAACAAGCTTTCAACACACCCTAG | Dre Rv | | CGCATCTTGAATCTCTGCATAC |
| Xtr Fw | | GAGACGGTCACTGGCAAGGA | Xtr Rv | | CGTCCAGCTCTTCAACGTTC |
| Gga Fw | | GTTATGCGACCGTCACCGCG | Gga Rv | | CGAACATGAACTTCTGATGC |
| **Riboprobes** | |  |  | |  |
| CRX Fw | | CCTTCCCGAGTCCAGAGTTC | CRX Rv | | AAGAGCCATAGCCCTGGCTG |
| CERKL Fw | | AAGCTGAGCTGAACGGAGATG | CERKL Rv | | TCAGATACAGCTGCATTTCACC |
| OTX2 Fw | | GACTGCGGCTCATACTTGACG | OTX2 Rv | | CATCCTCTCTATCATCCTCCTG |
| PAX6a Fw | | ATGGTTGCCAACAGTCAGACG | PAX6a Rv | | CTGTAGTCTGGGCCAGTATTG |
| **Validation of morpholinos** | | |  | |  |
| Exon 3 Fw | | CTCGCTGAT ATACAAGTGG ATG | Exon 5 Rv | | GAGAGGAAGTGCTGCTTGGAC |
| Exon 8 Fw | | ACCTGAAGATTGTGAACTGTCC | Exon 10 Rv | | CTGGTGTTTGGAGCCAGGCC |
| Intron 3 Fw | | AGGACAAGAACGGTTGCAACTC | Intron 3 Rv | | TAAATGCATGCAGAGATGGCAG |
| Intron 9 Fw | | GTCCATCATCTATAAGCACTAAG | Intron 9 Rv | | TTCACTCCTTCATTCTCTAGTC |
| Exon 11 Fw | | CATCAAGCACTTGAAGAGATACA | Exon 13 Rv | | TCCAAAGAGTGTGAGGAGCTG |
